# Characteristics for a tool for timely identification of palliative needs in heart failure: The views of Dutch patients, their families and healthcare professionals

**DOI:** 10.1177/1474515120918962

**Published:** 2020-05-05

**Authors:** Daisy JA Janssen, Stephanie MC Ament, Josiane Boyne, Jos MGA Schols, Hans-Peter Brunner-La Rocca, José MC Maessen, Marieke HJ van den Beuken-van Everdingen

**Affiliations:** 1 Department of Research & Development, CIRO, Horn, The Netherlands; 2 Department of Health Services Research, Care and Public Health Research Institute, Faculty of Health Medicine and Life Sciences, Maastricht University, The Netherlands; 3 Department of Patient and Care, Maastricht University Medical Centre (MUMC+), The Netherlands; 4 Department of Family Medicine, Care and Public Health Research Institute, Faculty of Health Medicine and Life Sciences, Maastricht University, The Netherlands; 5 Department of Cardiology, Maastricht University Medical Centre (MUMC+), The Netherlands; 6 Centre of Expertise for PC, Maastricht University Medical Centre (MUMC+), The Netherlands

**Keywords:** Instrument, end-of-life, palliative medicine, assessment, implementation, congestive heart failure

## Abstract

**Background:**

Palliative care can improve outcomes for patients with advanced chronic heart failure and their families, but timely recognition of palliative care needs remains challenging.

**Aim:**

The aim of this study was to identify characteristics of a tool to assess palliative care needs in chronic heart failure that are needed for successful implementation, according to patients, their family and healthcare professionals in The Netherlands.

**Methods:**

Explorative qualitative study, part of the project ‘Identification of patients with HeARt failure with PC needs’ (I-HARP), focus groups and individual interviews were held with healthcare professionals, patients with chronic heart failure, and family members. Data were analysed using the Consolidated Framework for Implementation Research.

**Results:**

A total of 13 patients, 10 family members and 26 healthcare professionals participated. Direct-content analysis revealed desired tool characteristics for successful implementation in four constructs: relative advantage, adaptability, complexity, and design quality and packaging. Healthcare professionals indicated that a tool should increase awareness, understanding and knowledge concerning palliative care needs. A tool needs to: be adaptable to different disease stages, facilitate early identification of palliative care needs and ease open conversations about palliative care. The complexity of chronic heart failure should be considered in a personalized approach.

**Conclusions:**

The current study revealed the characteristics of a tool for timely identification of palliative care needs in chronic heart failure needed for successful implementation. The next steps will be to define the content of the tool, followed by development of a preliminary version and iterative testing of this version by the different stakeholders.

## Introduction

The value of timely initiation of palliative care for people with chronic heart failure (CHF) has been shown.^1^ Indeed, palliative care interventions can improve quality of life, alleviate symptoms, improve patient satisfaction, increase documentation of care preferences and may decrease health service utilization.^2^ Nevertheless, we are still far away from routinely offering palliative care to patients with advanced CHF and their families.^3^ If referrals to palliative care are made, this is often very late in the course of the disease.^4^,^5^

Several barriers exist for timely and adequate provision of palliative care to people with CHF. These barriers include the misperception that palliative care is appropriate only at the end of life, false expectations concerning prognosis among both patients and healthcare professionals (HCPs), resulting in avoidance of discussions about likely future outcomes, prognostic uncertainty and lack of training of CHF clinicians in palliative care.^3^

CHF is characterized by an uncertain prognosis and highly individual disease trajectories.^6^,^7^ To facilitate timely recognition of patients with CHF in need of palliative care, an approach focusing on identification of palliative care needs seems to be more appropriate than the recognition of a poor prognosis.^8^ A few tools have been developed for this purpose, such as the Australian ‘Needs Assessment Tool: Progressive Disease – Heart Failure’ (NAT:PD-HF).^8^,^9^ Nevertheless, the usefulness of this tool was limited in a Dutch healthcare setting and CHF nurse specialists identified several barriers towards palliative care needs assessment, although they recognized the value of such a tool.^10^ Thus, a tool is needed that timely identifies palliative care needs in patients with advanced CHF, meeting the needs of Dutch patients, their families and HCPs. Therefore, the aim of the current qualitative study is to identify characteristics of a tool to timely recognize palliative care needs in CHF that are needed for successful implementation, according to patients, their families and HCPs in The Netherlands.

## Methods

### Design and ethics

This qualitative study is part of the project ‘Identification of patients with HeARt failure with palliative care needs’ (I-HARP). The aims of the I-HARP project are to develop and implement a tool to timely recognize palliative care needs in CHF and to develop a training for HCPs. For the current study, focus group interviews with HCPs, individual qualitative interviews with HCPs who were unable to attend a focus group, and individual interviews with patients with CHF, family members and bereaved family members were performed between October 2018 and April 2019. The medical ethical committee of the Maastricht University Medical Centre (MUMC+), Maastricht, The Netherlands reviewed the study protocol and concluded that the study did not require medical ethical approval as the study did not fall under the Medical Research Involving Human Subjects Act (2018-0638). Participants provided written informed consent. The study conforms with the principles outlined in the Declaration of Helsinki.^11^

### Participants

HCPs, patients, family members and bereaved family members were recruited in one general practice, two academic hospitals and two nursing homes. HCPs were purposively sampled and were eligible if they were clinicians caring for patients with CHF, including registered nurses, CHF nurse specialists, general practice-based nurse specialists, family physicians, cardiologists (in training), palliative care physicians and elderly care physicians. Patients and family members were informed about the study by their HCP and, if they agreed, they were contacted by a member of the research team and they received written information. Eligible patients were patients diagnosed with CHF and New York Heart Association (NYHA) class III or IV and were able to participate in qualitative interviews. Family members of patients with CHF NYHA class III or IV were eligible. Bereaved family members were included between six and 12 months after the death of the patient. Patients with congenital heart disease were excluded.

### Data collection

All individual and focus group interviews were performed by a member of the research team with training and experience in qualitative interview techniques. Topic lists, developed by the research team with backgrounds in cardiology, palliative care, elderly care medicine, and nursing, were used (see Supplemental Material online). The interview guides for patients and family members were pilot tested with two patients and one caregiver. Cards showing images that trigger feelings and attitudes were used as a probe during the interviews with patients, family members and bereaved family members to open up the conversation and to help them to express their actual experiences, emotions and wishes for desired care.^12^ If patients wished to do a couple interview with a family caregiver, this wish was respected. Each interview started with an introduction, including the aim of the meeting, the research project and the rules of the interview. Participants also gave written permission for audio recording. A moderator and an observer conducted each focus group. Interviews were recorded and transcribed verbatim. Field notes were made after each interview. A member check was performed by submitting an interview summary to each respondent for approval, and to check accuracy and credibility of the data.

### Data analysis

Data were analysed using Nvivo version 12 PRO. Data analyses started directly after the first interview using direct content analysis.^13^ Researchers independently coded each group of stakeholders (patients, family members and bereaved family members (SMCA, LvH, LH); HCPs (SMCA, WE, IC)). All interviews were coded by the central coder (SMCA). For the less rich interviews – in the opinion of the coding team – dependent coding of the transcript was performed. Agreement on the coding was reached during consensus meetings. Initially, data of the patients, family members and bereaved family members on the one hand and HCPs on the other hand were analysed separately to make sure that both perspectives were heard.

To explore the desired tool characteristics needed for successful implementation, the data concerning the HCPs were analysed deductively using the Consolidated Framework for Implementation Research (CFIR).^14^ Data were categorized into the CFIR’s domain ‘intervention characteristics’. An inductive coding approach was used to explore and identify desired tool characteristics from the patients’, family members’ and bereaved family members’ perspective. After data saturation level was reached, the revealed constructs were coded deductively into the CFIR’s construct ‘intervention characteristics’.

## Results

### Participants

In total, 13 patients (four recruited in primary care, seven recruited in hospitals and two recruited in nursing homes), seven family members and three bereaved family members were interviewed at home (*n* = 17), in the nursing home (*n* = 3), the university (*n* = 1) or the hospital (*n* = 2). Three eligible patients refused to participate due to their actual condition; and one patient was abroad for an unknown time. Two focus group interviews were held in two different hospitals. One group included six HCPs: two cardiologists, one heart failure nurse specialist, one team leader, one registered nurse and one certified nurse assistant. The other group included eight HCPs: two cardiologists, two heart failure nurse specialists, one team leader, one palliative care specialist/consultant, two registered nurses. One focus group interview with four participants was held in a nursing home: three registered nurses and one medical doctor (Table 1). Four family physicians, two general practice-based nurse specialists, one elderly care physician and one palliative care consultant working in the hospital were interviewed by phone while they were unable to attend a focus group meeting. The mean duration of the individual interviews with patients was 61 (range 26–92) min, for family members and bereaved family members 67 (range 51–79) min and of the individual interviews with HCPs 45 (range 30–54) min. Mean duration of the focus group interviews was 104 (range 99–110) min.

**Table 1.** Characteristic of participants.

**Patients *n*=13**	**Age in years, median (range)**	71 (51–89)
	**Male, *n* (%)**	8 (62)
**Family members *n*=7**	**Age in years, median (range)**	71 (62–86)
	**Male, *n* (%)**	0 (0)
**Bereaved family members *n*=3**	**Age in years, median (range)**	57 (43–73)
	**Male, *n* (%)**	0 (0)
**Healthcare professionals *n*=26**	**Age in years, median (range)**	46 (25–66)
	**Male, *n* (%)**	7 (27)
	**Registered nurses, *n* (%)**	6 (23)
	**Certified nurse assistant, *n* (%)**	1 (4)
	**Heart failure nurse specialists, *n* (%)**	3 (12)
	**General practice-based nurse specialists, *n* (%)**	2 (7)
	**Family physicians, *n* (%)**	4 (15)
	**Cardiologists, *n* (%)**	4 (15)
	**Palliative care specialist/consultants, *n* (%)**	2 (7)
	**Elderly care physician, *n* (%)**	1 (4)
	**Medical doctor (nursing home), *n* (%)**	1 (4)
	**Team leader, *n* (%)**	2 (7)

### Tool characteristics

**Figure 1.**
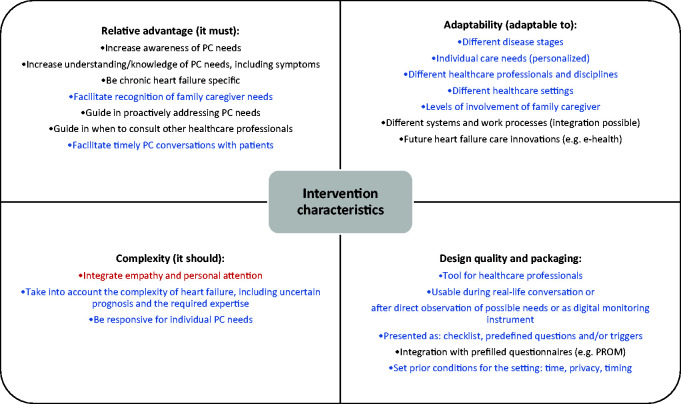
Desired intervention characteristics for timely identification of palliative care needs in chronic heart failure. Tool characteristics categorized into relative advantage, adaptability, complexity and design quality and packaging. Shown in black: reported by healthcare professionals. Blue: reported by healthcare professionals and patients/family caregivers. Red: reported by patients/family caregivers. PC: palliative care; PROM: patient related outcome measure.

Content analysis revealed desired tool characteristics for successful implementation in four constructs: relative advantage, adaptability, complexity, and design quality and packaging. These are summarized in Figure 1 and described below.

#### Relative advantage

*Relative advantage* refers to the perception of patients, families and clinicians concerning the characteristics needed to experience benefits from using the tool.^14^

HCPs indicated that a relative advantage would be if the tool increased awareness, understanding and knowledge concerning palliative care needs in CHF. HCPs working in primary care or nursing homes wanted more guidance in proactive recognition of palliative care needs in advanced CHF, including more understanding of frequent symptoms (Table 2, quote 1). HCP mentioned that they needed guidance in how to address palliative care needs, including when to refer to other HCPs. They discussed the need for a CHF specific tool. Indeed, some HCPs mentioned that an existing generic tool with triggers for palliative care, such as the Supportive and Palliative Care Indicators Tool, is too broadly oriented for use in CHF, without signs of a poor prognosis CHF. This may result in not identifying CHF patients with palliative care needs. Respondents suggested that a tool should not only facilitate recognition of palliative care needs of patients, but also of their family caregivers, for example a need for information (Table 2, quote 2).

**Table 2.** Quotes of participants.

Quote	
**1**	*As physician we are trained in addressing the current request for help. That is where the patient has a request for help, but problems or issues that may arise in the future, the patient doesn’t know that. As family physician you need to anticipate on those problems and that is a complete other way of thinking. Not reactive, but what happens frequently in heart failure? If there is anxiety, you can do this.* (Family physician)
**2**	*But you know … heart failure, what is it exactly? I still don’t know. I still don’t know what heart failure exactly is.* [Interviewer:] *Has that never been told?* [Family caregiver:] *No, not in such words. Maybe I should have myself … I always joined him. Then the situation was explained about the tablets etc. Maybe I should just have asked it once. What is it now exactly? Because I knew he didn’t like that, I never did. If I had asked* [name of heart failure nurse specialist], *she would have explained it to me in detail. But now I still don’t know what it is.* (Bereaved family member)
**3**	*If you can use it to prepare your outpatient clinic, to be able to introduce certain difficult topics, then it doesn’t need to be so hard, but you can see whether there is a desire to talk about a certain topic. Then there is an opening. That’s what you are looking for, an opening. And awareness for yourself.* (Cardiologist)
**4**	*It isn’t from one moment to the next, but if you can refer to ‘you know, what we discussed last time, are there things that you keep thinking about?’ and at a certain moment you get to using the words ‘PC’. But it is so loaded because palliative is confused with terminal.* (Family physician)
**5**	*The moment that they say in the hospital ‘we can’t treat you any more’. We knew that. It used to be once each half year infusion therapy, it became every three weeks, every two weeks. We knew that, we couldn’t go on like that. But at that moment, that infusion, we can’t treat you anymore, you can go home … I didn’t even know the word PC … * (Patient)
**6**	*We have an interdisciplinary PC meeting and discuss a patient if the answer to the Surprise Question is ‘no’. Then you expect a patient to be in the palliative stage of the disease. That can’t be the cut-off point. I think they are longer in the palliative stage of the disease if they are chronically ill*. (General practice-based nurse specialist)
**7**	*You grow towards something and at a certain moment you say, and that can be because of a situation, I don’t want resuscitation anymore … and then at a certain moment you accept more and more, or you accept that new phase.* (Patient)
**8**	*You don’t know how a patient will react. He can show almost no response, until you* [nurse points at cardiologist] *leave and then we have a patient who is completely in distress.* (Nurse)
**9**	*Who do you need to go to? Then you find yourself in a maze, I don’t know any more.* (Patient)
**10**	*My husband absolutely didn’t want to talk about it. And he also didn’t want me to have conversations with the nurses or doctor alone. That made it very difficult. And that made me feel very lonely.* [ … ] *Once I did talk with them and he heard that. Then he said: ‘*[name]*, I don’t want you to talk with them behind my back’.* (Bereaved family member)
**11**	*We have to search for something, not static, but dynamic. There is so much technology available.* (Team leader, cardiology ward)
**12**	*People who become really old while having severe heart failure, that’s something from the last 15 years. But in our idea of how people die, we don’t think of heart failure. And the patients are patients who other than an oncology patient often years ago had a myocardial infarction and know their limitations for years. And don’t have the feeling that there is something to report.* (Family physician)
**13**	*Struggling, but also fighting for it. And they want to live, I feel that as a message. But in the end, the terminal phase or deterioration. For me, that’s the most difficult part, the grey area; they come to your outpatient clinic, they are informed, maybe not the first conversation, but the second one* [ … ]*. And then finally, suddenly you see them deteriorate fast*. (CHF nurse specialist)
**14**	*Further, I think that protocols are good, but this, in particular, is something that requires a real personal approach and tailored care. Everybody is different.* (Cardiologist)
**15**	*I think they* [HCPs] *should have brought it up proactively. I am one of the patients who knows very well what’s going on and I keep track of it very well myself*. (Patient)
**16**	*For me, it’s difficult to have such a structured conversation. Often you know people longer, including their home situation. Then it’s difficult to address this per item. You can learn a lot in a conversation, especially when you visit the patient at home, you see things and also the family*” (General practice-based nurse specialist)
**17**	*I think that it should provide guidance, not be a checklist. *(Cardiologist) *And include a few questions to start the conversation.* (Nurse, hospital setting)
**18**	*When I started here with PC, I often said, I need a sort of checklist for care needs, to complete who is addressing this or that. That works more easily. Now it’s based on my experience or what the patient or family asks. That’s not always correct or I can overlook things.* (General practice-based nurse specialist)
**19**	*I can’t see myself using a checklist. I see the client and what’s normal and is functioning normal. Can he speak complete sentences or is it just one word what he mentions? I have my own checklist, my own backpack.* (Nurse, nursing home)
**20**	*When they are recently discharged from the hospital. Then you have a trigger (to discuss palliative care). If you just visit them spontaneously, I also find it a difficult conversation, when there is no trigger.* (Family physician)

PC: palliative care; HCP: healthcare professional.

HCPs working in a hospital mentioned that an essential advantage would be if the tool facilitated them to initiate conversations about palliative care needs (Table 2, quote 3). People with CHF, caregivers and HCPs mentioned that the choice of specific words to timely discuss palliative care needs is essential. HCPs in primary care and the hospital need a tool facilitating them in finding the right words and questions (Table 2, quotes 4 and 5).

### Adaptability

*Adaptability* relies on a definition of the ‘core components’ (the essential and indispensable elements of the tool itself) versus the ‘adaptable periphery’ (adaptable elements, structures and systems related to the tool and organization into which it is implemented) of the tool.^14^

Patients, family caregivers and HCPs mentioned that the tool needs to be adaptable to different stages of the disease and facilitate early identification of palliative care needs. HCPs reflected on existing tools and some felt that the focus of the Surprise Question (‘Would I be surprised if this patient died in the next 12 months?’) on the last year of life is not appropriate for chronic life-limiting illnesses such as CHF (Table 2, quote 6). In addition, HCPs mentioned that they experience different levels of involvement during the journey of the patient with CHF. This corresponds with the finding that patients with CHF experienced different disease stages with different levels of acceptance and probably different care needs (Table 2, quote 7). HCP reported that many healthcare organizations and professionals are involved in identification of palliative care needs and perceived difficulties in providing interdisciplinary palliative care (Table 2, quote 8). Therefore, a tool must be suitable for repeated use for the same patient in different settings (e.g. during a home visit, at the outpatient clinic or in a nursing home) by the same or another healthcare professional. Some patients with CHF and their family members reflected on the lack of clarity regarding whom to consult with specific disease related issues (Table 2, quote 9).

According to patients with CHF and their family members, personal preferences of the patient and family member regarding whether and to what extent to involve the family member in palliative care must be taken into account (Table 2, quote 10). HCPs also mentioned that they need a tool which can be used with the patient alone but also in the presence of the family member.

HCPs in all settings mentioned that the integration of the tool into existing registration systems and current work processes (e.g. advance care planning discussions in nursing homes, electronic patient records of family physicians) may stimulate the uptake of the tool in daily practice. HCPs mentioned that the tool needs to be adaptable to future innovation in the context of cardiology, for example, digital monitoring and the increased use of tablets or smartphones (Table 2, quote 11).

### Complexity

*Complexity* refers to perceived difficulty of the intervention, reflected by intervention type, duration, scope, radicalness, disruptiveness, centrality, and intricacy and number of steps required to implement.^14^

According to patients with CHF and their family members, elements such as empathy, respect and personal attention to the patients need to be part of the method to identify palliative care needs.

Another complex element HCPs mentioned is the complexity of the disease itself. HCPs in all settings and patients mentioned the specific patient characteristics of CHF, such as that their actual care need is difficult to comprehend because, for example, patients have their symptoms and limitations for years, accept these and do not mention these spontaneously (Table 2, quote 12). HCPs working in the hospital reported the challenge of the uncertain prognosis in relation to the, for them, hidden palliative care needs. They referred to this as the grey area (Table 2, quote 13).

HCPs, patients and family members mentioned the importance of personalized identification of palliative care needs (Table 2, quote 14).

### Design quality and packaging

*Design quality and packaging* includes how the intervention should be bundled, presented and assembled and how accessible the tool is for users.^14^

Both HCPs and patients mentioned that it is the HCP’s responsibility to identify palliative care needs (Table 2, quote 15). People with CHF stated that palliative care needs can be identified during a real-life conversation, by patient’s observation during regular care delivery and by using a digital monitoring instrument. One patient mentioned the use of a checklist for palliative care signals. Frequently, patients mentioned that they would withhold information if HCPs use checklists or focus on the computer. Patients added that it is important that HCPs listen carefully to them and that it is important to let the patient talk. Both patients and HCPs believed that palliative care conversations would be easier if they happened more frequently, so that patients knew what to expect and had time to think about it. HCPs working in primary care recognized the need for open conversations (Table 2, quote 16).

HCPs working in the hospital mentioned the need for guidance in conversations addressing palliative care needs (Table 2, quote 17). HCPs also mentioned that palliative care conversations could be facilitated if patients completed a questionnaire at home to explore problems in different palliative care domains and brought it with them to the consultation.

While some HCPs said that a checklist would not be used in practice, other HCPs believed that a checklist would help them during the early learning stage or later if they are in doubt (Table 2, quote 18). Especially in nursing homes, observation seemed the most preferred method to identify palliative care needs (Table 2, quote 19).

Other HCPs mentioned the need for triggers to plan conversations, such as the second admission for decompensated CHF. HCPs from all settings mentioned that they need triggers or a signal from another HCP (for example the cardiologist) to initiate a conversation about palliative care needs (Table 2, quote 20). Patients and caregivers indicated that time and privacy are required conditions for palliative care conversations. They preferred these conversations a few weeks after discharge at the outpatient clinic or at home.

## Discussion

### Key findings

The present study is the first to identify desired characteristics of a new Dutch tool to assess palliative care needs in CHF, as identified by patients, their families and clinicians. These findings are needed to design such a tool with a high likelihood of successful implementation. We addressed four CFIR constructs in this study. The current study also shows the complexity of palliative care needs assessment in this population, with different stakeholders showing different needs.

### Relative advantage

We found several characteristics needed to experience advantage of the use of a tool. *First*, HCPs requested a tool which could increase awareness concerning palliative care needs. The recently published expert position statement ‘Palliative care for people living with heart failure’ from the European Association for Palliative Care describes the importance of a needs assessment based approach for early initiation of palliative care for patients with advanced CHF and states that the reliance on prognostic triggers is ineffective.^15^ Palliative care needs hardly correlate with prognosis, and prognostic uncertainty can be a barrier to timely assessment of palliative care needs.^15^ To facilitate this timely assessment, a tool should be easy to use in a broad range of patients with advanced CHF during regular clinical care.

So, *second*, the tool should facilitate conversations about palliative care needs, while using language which is not confronting. Thus a Dutch tool should differ from the previously tested NAT:PD-HF^9,10^ in this aspect: it should be a tool to help HCPs with timely communication about palliative care needs. This is needed so that the tool is able to be used in patients who do not recognize their illness as possibly life-threatening as well as for HCPs with limited expertise in palliative care discussions. This seems especially important in a disease such as CHF. A recent Canadian qualitative study showed that patients did not recognize acute decompensated heart failure as a serious event. They saw recurrent hospitalizations as normal, without recognition of the progressive nature of the disease and threat of mortality.^16^ Not-confronting language is also important for HCPs, who reported a need for support in initiation of palliative care needs conversations. A survey found that half of the clinicians taking care of patients with CHF reported reasons not to discuss palliative care, such as their own discomfort, the feeling that patient or family were not ready, or fear of destroying hope.^17^

*Third*, HCPs reported the need for increase of knowledge concerning palliative care needs. The previously mentioned survey also found that 30% of clinicians had a low or very low level of confidence in providing palliative care or palliative care discussions.^17^ So, training in palliative care needs and guidance in actions, which is also not included in the NAT:PD-HF, is probably needed for successful implementation of a tool to recognize palliative care needs.^10^

*Fourth*, the need for a CHF specific tool was mentioned. Although participants did not elaborate on this aspect, it is reasonable to assume that the previously discussed disease trajectory of CHF, as well as CHF specific palliative needs, require attention in a tool. For example, differences might exist in symptom burden between patients with CHF and patients with other life-limiting diseases.^18^ Also disease-specific aspects are relevant in palliative care conversations, such as, for example, the discussion of deactivation of an implantable cardioverter defibrillator.^19^ On the other hand, patients with CHF often have multiple morbidities, which can also result in palliative care needs.^20^

*Fifth*, the tool should also pay attention to the needs of the family member. Family members have key roles in the care for patients with advanced CHF and often neglect their own needs and personal health.^21^ So, proactively asking family members to reflect on their needs is essential. In our study, family members reported a need for information. This is in line with findings of a systematic review, revealing the need for information as the most important concern from family caregivers, especially to be prepared for future challenges, including making difficult decisions.^21^

### Adaptability

The need for adaptability in different ways was also mentioned. For example, a tool should be adaptable to different disease stages, different HCPs and different settings. Multiple HCPs are involved in care for patients with advanced CHF.^20^ Ideally, a tool should also facilitate collaboration between these HCPs, while lack of collaboration is an important barrier for palliative care conversations with patients with CHF.^22^ For example, a recent review showed that cardiologists stated that these conversations should be done by the family physician because of the long-term relationship with the patient, while some family physicians found that these conversations should be performed by the cardiologist, being the expert in CHF.^22^ On the other hand, HCPs working in cardiology reported to require training to be able to address palliative care needs. Limited knowledge of community nurses about the role of CHF nurse specialist can also delay initiation of palliative care.^22^ Although attention to family members is important, personal preferences of the patient and family members regarding whether and to what extent to involve the family members should be addressed and respected. This is especially important as patients with CHF and family members may differ in their perspectives on the future with CHF, resulting in different needs for communication about palliative care.^23^ Therefore, taking into account individual family dynamics is important in providing palliative care.^22^ Finally, the integration of a tool in different current systems and work processes as well as in future innovations, such as, for example, e-health, is needed.

### Complexity

Participants reflected on the perceived difficulty of a tool to timely identify palliative care needs in CHF. They mentioned the need for a personal approach as well as the complexity of CHF, including the uncertain prognosis. This is in accordance with previous literature, showing that the uncertain prognosis limits timely initiation of advance care planning in CHF.^22^ On the other hand, a personal approach, for example, by creating more time for conversations, being able to clarify what a patient wants and does not want to know, and knowing personal preferences and values facilitates timely advance care planning.^22^

### Design quality and packaging

One aspect that both patients and HCPs agreed upon is that HCPs are responsible for identifying palliative care needs. Whether they should do this during a conversation, by observation, by using a digital monitoring instrument or by a checklist remains unclear. Patients reported that having the opportunity to talk and feel listened to is paramount. HCPs, in turn, reported the need for guidance in conversations about palliative care needs. So, ideally a tool should facilitate these conversations by guiding HCPs in stimulating patients to talk about their actual needs.

### Methodological considerations

An important strength of the current study is the involvement of different stakeholders, including different HCPs. This is especially important, as a previous review has shown that attitudes regarding palliative care conversations may differ between disciplines.^22^ Another strength is the prospective use of the CFIR framework, an evidence-based framework for implementation of practice-transforming initiatives.^24^

The study also has limitations, which should be considered. *First*, this study shows characteristics of a tool as identified by Dutch patients, family members and HCPs. Important international differences exist in palliative care,^25^,^26^ so findings may not be directly applicable in other countries. Nevertheless, as discussed above, many emerging constructs in our interviews are also shown in other (international) studies. *Second*, we have found characteristics in only four constructs of the CFIR’s domain ‘intervention characteristics’. So future studies should explore other CFIR constructs, such as intervention source, evidence strength and quality, trialability and costs. *Third*, male family members and male HCPs were underrepresented in the current study. *Finally*, the interviews were moderated by a member of the research team. This could have introduced bias by unknowingly directing the discussion. Nevertheless, the moderator was a researcher without clinical background and her role was to facilitate the discussion and not to take part in the discussion. An advantage of this approach is that she was able to ask more in-depth questions, while she was simultaneously involved in data-analysis.

## Conclusions

By using the CFIR framework, this study identified characteristics of a tool to assess palliative care needs in CHF, for successful implementation, according to patients, their families and HCPs in The Netherlands. Thus, it should create awareness concerning palliative care needs in both patients and family members, should be easy to use in different disease stages and should facilitate conversations about palliative care needs. Complexity of the disease and personal preferences must be taken into account. Training in palliative care needs and guidance in conversations about palliative care as well as actions to address palliative care needs seem to be required for successful implementation. The next steps will be to define the content of the tool, followed by development of a preliminary version and iterative testing of this version by the different stakeholders.

## Implications for practice

- A tool may support recognition of palliative care needs;
- A tool should help in creating awareness of needs;
- A tool should facilitate palliative care conversations;
- Guidance is needed for successful implementation.
